# Microvessel stenosis, enlarged perivascular spaces, and fibrinogen deposition are associated with ischemic periventricular white matter hyperintensities

**DOI:** 10.1111/bpa.13017

**Published:** 2021-09-19

**Authors:** Austyn D. Roseborough, Berk Rasheed, Youngkyung Jung, Kevin Nishimura, William Pinsky, Kristopher D. Langdon, Robert Hammond, Stephen H. Pasternak, Ali R. Khan, Shawn N. Whitehead

**Affiliations:** ^1^ Vulnerable Brain Laboratory Department of Anatomy and Cell Biology The Schulich School of Medicine & Dentistry The University of Western Ontario London Ontario Canada; ^2^ Michael G DeGroote School of Medicine McMaster University Hamilton Ontario Canada; ^3^ Department of Physiology and Pharmacology The Schulich School of Medicine & Dentistry The University of Western Ontario London Ontario Canada; ^4^ Department of Pathology and Laboratory Medicine The Cumming School of Medicine The University of Calgary Calgary Alberta Canada; ^5^ Department of Pathology and Laboratory Medicine The Schulich School of Medicine & Dentistry The University of Western Ontario London Ontario Canada; ^6^ Department of Clinical Neurological Sciences Robarts Research Institute The Schulich School of Medicine & Dentistry The University of Western Ontario London Ontario Canada; ^7^ Department of Medical Biophysics The Schulich School of Medicine & Dentistry The University of Western Ontario London Ontario Canada

**Keywords:** fibrinogen, periventricular infarct, small vessel disease, stenosis, white matter disease, white matter hyperintensity

## Abstract

Periventricular white matter hyperintensities (pvWMH) are neuroimaging abnormalities surrounding the lateral ventricles that are apparent on magnetic resonance imaging (MRI). They are associated with age, neurodegenerative disease, and cerebrovascular risk factors. While pvWMH ultimately represent a loss of white matter structural integrity, the pathological causes are heterogeneous in nature, and currently, cannot be distinguished using neuroimaging alone. pvWMH could occur because of a combination of small vessel disease (SVD), ependymal loss, blood–brain barrier dysfunction, and microgliosis. In this study we aimed to characterize microvascular stenosis, fibrinogen extravasation, and microgliosis within pvWMH with and without imaging evidence of periventricular infarction. Using postmortem neuroimaging of human brains (*n* = 20), we identified pvWMH with and without periventricular infarcts (PVI). We performed histological analysis of microvessel stenosis, perivascular spaces, microgliosis, and immunohistochemistry against fibrinogen as a measure of serum protein extravasation. Herein, we report distinctions between pvWMH with and without periventricular infarcts based on associations with microvessel stenosis, enlarged perivascular spaces, and fibrinogen IHC. Microvessel stenosis was significantly associated with PVI and with cellular deposition of fibrinogen in the white matter. The presence of fibrinogen was associated with PVI and increased number of microglia. These findings suggest that neuroimaging‐based detection of infarction within pvWMH may help distinguish more severe lesions, associated with underlying microvascular disease and BBB dysfunction, from milder pvWMH that are a highly frequent finding on MRI.

## Graphical abstract

White matter fibrinogen deposition is associated with post‐mortem MRI evidence of periventricular infarcts, arteriole stenosis and increases in microglia.

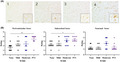

## 1 INTRODUCTION

White matter hyperintensities (WMH) are a commonly reported neuroimaging finding apparent on magnetic resonance imaging (MRI) that are associated with age, Alzheimer's dementia, and cerebrovascular risk factors [1, 2]. The pathology of WMH is heterogeneous in nature but core features include rarefaction of myelin and axon structure, microvascular disease, and glial cell activity [3, 4, 5]. WMH are often considered imaging features of cerebral small vessel disease (SVD), where pathology of the microvasculature involving stenosis, lipohyalinosis, and collagenosis of the arterioles and venules contributes to chronic ischemia of the white matter [6, 7, 8, 9, 10]. WMH are usually detected in two primary categories: periventricular (pvWMH), surrounding the lateral ventricles, and deep WMH located within the subcortical white matter. pvWMH are highly prevalent on FLAIR and T2‐weighted MRI sequences in aged individuals, appearing in the majority of adults over the age of 60 [11]. They can present as a thin “halo” surrounding the ventricles, “caps” at the frontal or occipital horns, or, in more severe instances can extend outward into the subcortical white matter [12]. In cognitively normal aged subjects, pvWMH are associated with global cognitive decline, executive dysfunction, decreased processing speed, and gait impairments [13, 14, 15]. Frontal pvWMH in particular can be strategically located in white matter tracts such as the superior longitudinal and uncinate fasciculi; disruption of which may mediate their cognitive consequences [16].

Despite their high prevalence, and although they have been investigated frequently in neuroimaging studies, the development of pvWMH from a pathological perspective remains poorly understood. While deep WMH are presumed to be of vascular origin, pathological reports of pvWMH are less consistent. Imaging of pvWMH alone is insufficient to determine the underlying etiology, and it is possible that both ischemic and nonischemic mechanisms contribute to their development. Some studies have reported myelin pallor and ependymal denudation to be the primary features of mild pvWMH, with minimal involvement of vascular pathology [17, 18, 19, 20]. Others report vascular alterations such as atherosclerosis [21], venous collagenosis [22], and enlargement of the perivascular space suggestive of an SVD‐mediated pathology [5]. These inconsistencies limit the clinical interpretation of pvWMH, which is further complicated by the wide spectrum of lesion severity, and the inability to predict underlying etiology using neuroimaging alone.

One possible consequence of underlying vascular disease implicated in ischemic pvWMH involves the breakdown of the BBB and extravasation of serum proteins into the white matter. It has been proposed that chronic hypoperfusion because of microvessel stenosis can result in BBB dysfunction via loss of pericytes and damage to the endothelial integrity [23]. Support for the mechanistic link between vascular disease and BBB dysfunction comes from recent imaging and pathological studies. Imaging studies have confirmed BBB breakdown within the white matter of subjects with WMH in association with cognitive deficits [24, 25]. Additionally, pathological studies have reported extravasation of the serum protein fibrinogen into both white matter and cortical tissue [26, 27], and it is known that the tight regulation of plasma protein transport by the BBB is altered with aging, resulting in an increase in leakiness [27, 28, 29]. Once it has crossed the BBB, fibrinogen is suggested in *in vitro* and in preclinical studies to impair remyelination and promote the accumulation of activated microglia [30, 31, 32]. With respect to human studies, it is not yet well understood what role fibrinogen plays in the development of white matter pathology and whether this involves significant microglial activity. Therefore, the potential effects of fibrinogen on the white matter and the homeostasis of its supportive glial network warrants further study in the context of WMH with underlying microvascular disease.

Determining vascular contributions to pvWMH on MRI imaging may be aided by the presence of periventricular infarcts (PVI) within the hyperintense tissue. Higher imaging resolution caused by advancements in MRI field strengths can detect small lesions of ischemic nature within pvWMH [33]. We have previously reported that the presence of these fluid filled cavities in brains with cerebrovascular disease are a significant contributor to the hyperintense signal in both the surrounding lesion and normal‐appearing white matter [33]. Of the few studies that have considered PVI separately from pvWMH, associations with vascular stenosis and collagenosis have been reported [22, 34], but whether there are resulting impairments to the BBB and glial cell activity is not well understood. To address this gap, we sought to investigate whether the presence of PVI, as an indicator of ischemic severity in pvWMH, is associated with microvessel stenosis and enlarged perivascular spaces characteristic of SVD. Our second aim was to investigate BBB dysfunction and microgliosis as potential mechanistic correlates of ischemic pathology in PVI. To do this, we investigated the white matter of a heterogeneous collection of human brain specimens with postmortem MRI evidence of pvWMH in combination with pathological analysis. This direct comparison of imaging and pathology allowed for the identification of fibrinogen deposition within clinically relevant MRI imaging of pvWMH and represents the first study, to our knowledge, reporting an association between BBB dysfunction and microgliosis in PVI across multiple diagnostic categories.

## 2 METHODS

### 2.1 Specimen selection

Specimens were chosen for MRI using neuropathological diagnosis and blinded to clinical diagnosis. Brains were stored in 10% formalin for up to 15 years. A coronal section from the frontal lobes, where all three frontal regions: superior, middle, and inferior were present, of 25 brains was selected for imaging. The final selection included 20 specimens with the following neuropathological diagnoses: five normal, five AD, five with features of cerebrovascular disease (CVD), and five with both AD and features of CVD. CVD specimens were grouped based on neuropathological reports that included reports of CVD such as subcortical infarcts, microinfarcts, and vasculopathy since there were rarely diagnoses that were the exact same in terms of CVD manifestation.

### 2.2 Imaging

All scans of postmortem tissue were performed at Western's Centre for Functional and Metabolic Mapping on a 7T Scanner (Siemens) following a previously described imaging protocol [33]. Briefly, frontal coronal sections were selected from each fixed brain and immersed in Galden HT‐270 perfluorinated fluid in a custom‐stacking device for imaging. T1‐weighted images from a MP2RAGE sequence, with an extended T1 time to improve contrast, and FLAIR images from a T2 SPACE sequence were acquired.

### 2.3 WMH assessment

Ratings and intensity quantification of WMH was performed blinded to neuropathological diagnosis and histological data. The MRI slice that corresponded to the coronal surface of the sectioned tissue was used for WMH rating. Periventricular WMH (pvWMH) were assessed on the FLAIR sequence and rated as either mild (extending outwards into <1/3 of the subcortical white matter) or moderate (>1/3 but <2/3 of the subcortical white matter involved). There were no specimens with extension into >2/3 of the depth of the subcortical white matter on the coronal section assessed. Focal hyperintensities that were present in deeper subcortical white matter, and not directly connected to the lateral ventricles were seen in 3/20 specimens and were not factored into the rating. The presence of periventricular infarction was evaluated using the T1‐weighted image and was defined as a periventricular fluid filled space, often located within the pvWMH, and confirmed histologically in order to exclude enlarged perivascular spaces that can be seen on T1‐weighted imaging [1]. We did not consider microinfarcts that were not visible on T1 imaging in order to limit analysis to lesions that could be appreciated on MRI clinically.

### 2.4 Tissue processing

Areas of periventricular white matter were sampled for tissue selection based on the MRI findings with the assistance of neuropathologists (KL & RH). Histological blocks were obtained from brain tissue that included the periventricular white matter, subcortical white matter, and cortical tissue; and placed into histological cassettes. One block from both the left and right hemispheres were sampled and stored in 10% neutral buffered formalin prior to histological processing.

### 2.5 Histology

All staining was performed by the Department of Pathology and Laboratory Medicine, University Hospital, and the Molecular Pathology Facility, Robarts Research Institute, in London, Canada. Tissue was paraffin embedded and sectioned at 8 μm thickness prior to staining with 0.1% Luxol Fast Blue (LFB) in 95% alcohol for 2 h at 60°C. Tissue was stained with *Ricinus communis*‐I (RCA) Lectin (Vector Labs, Brockville, Canada, 1:10,000) prior to Movat's pentachrome staining, the slides were placed in 0.5% periodic acid for 15′ caused by over‐fixation. The following steps were taken: Alcian blue solution 20′, running water wash 5′, Alkaline alcohol 5′ at 58°C, running water wash 5′, Verhoeff's Haematoxylin 5–15′, tap water wash, 1.4% ferric chloride, 95% ethanol 5′, running water wash, Acid fuschin solution 5′, rinse in water, rinse in 0.5% aqueous glacial acetic acid, differentiate in 5% aqueous phosphotungstic acid 15′, rinse in 0.5% aqueous glacial acetic acid, rinse three times with absolute alcohol for 2′ total, alcoholic saffron solution 5′, dehydrated three times with absolute alcohol.

### 2.6 Immunohistochemistry

Tissue was cryosectioned at 4 μm onto microscope slides (VWR International, Radnor, United States) and stained with monoclonal mouse anti‐smooth muscle actin (Agilent Dako, Mississauga, Canada, IR611, 1:500), polyclonal rabbit anti‐human fibrinogen (Agilent Dako, Mississauga, Canada, A0080, 1:200).

### 2.7 Histological assessment

Histological assessments were performed blind to neuropathological diagnosis and MRI imaging of SVD markers. *Stenosis and Perivascular Spaces:* Sclerotic index (SI) and PVS volume were calculated using a previously described method [35]. Measurements were taken at the maximum diameter of vessels that were not perfectly round. All periventricular vessels were located within 0.5 cm of the ependymal lining and subcortical vessels were in the white matter outside of this boundary. Movat's Pentachrome was used to distinguish components of the vascular walls and measure vascular diameter. Arterioles were distinguished from venules using smooth muscle actin staining to detect concentric rings of smooth muscle cells in the tunica media of arterioles. Vessels were further separated in the small (<50 µm), medium (50 < IntD < 200), and large (>200 µm) categories based on external diameter. Three vessels were measured for each size category in each region (periventricular & subcortical). *Fibrinogen Rating Scale:* Fibrinogen ratings were performed blinded to the neuropathological status of the specimen and was scored according to the most severe area present in a histological block. Cortical intraneuronal fibrinogen accumulations were rated according to a previously published scale: 0 = none, 1 = isolated, 3 = frequent (approximately 50% of neurons), 4 = very frequent (greater than 50% of neurons) [27]. Intraglial fibrinogen accumulation was defined as intracellular fibrinogen staining surrounding nuclei within the white matter which would represent oligodendrocytes, microglia, and astrocytes. Intraglial accumulations were rated as follows: 1 = none, 2 = isolated, 3 = frequent (approximately 50% of glia), 4 = very frequent (greater than 50% of glia). *Interstitial fibrinogen:* To capture interstitial staining of fibrinogen images were taken at 10x magnification from the pvWM of medium and large venules and arterioles (3 each). ROIs were drawn to encompass the area around each vessel, with a width of 1cm and excluding the perivascular space devoid of tissue. The percentage area of fibrinogen was quantified in the perivascular white matter using Image J with a threshold of 235. The percentage area coverage of nearby white matter without vessels in the field of view was calculated in order to normalize the background intensity. *Microglia Count:* Microglia cell numbers were generated using Image J with the following parameters optimized to distinguish them from endothelial cells: Threshold 130, Size 200–1200, and Circularity 0–0.5.

### 2.8 Statistics

All statistical analyses were completed with Graphpad Prism 8 software. Pearson's correlations values are reported for analysis of histological metrics. Group comparisons were performed using either unpaired Student's *t*‐test, One‐way ANOVA with Tukey's post‐hoc test, or Kruskal–Wallis test with Dunn's post‐hoc test with a significance value of *p* = 0.05.

## 3 RESULTS

**FIGURE 1:**
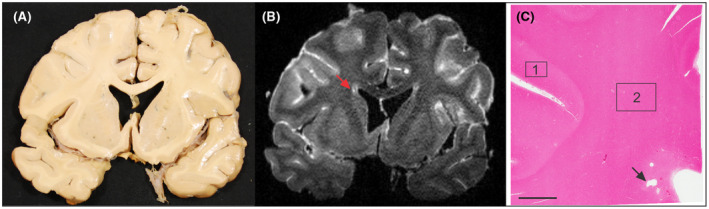
Example images of postmortem specimens and MR imaging that guided tissue blocking. (A) Coronal section of postmortem tissue. (B) FLAIR image with identification of pvWMH and fluid filled PVI (red arrow). (C) H&E stained tissue block corresponding to the region identified in B. Periventricular tissue was identified as tissue within 0.5 cm of the ependymal lining. Box 1 and 2 indicate examples of cortical tissue and subcortical white matter, respectively. Scale bar indicates 5 mm

Twenty brains were included in the final analysis: five normal brains, five Alzheimer's disease, five with pathological reports of cerebrovascular disease, and five with both Alzheimer's disease and cerebrovascular disease findings. Demographic and pathological data for specimens are reported in Table 1. The average age of specimens at time of death was 72.9 (11.7). Postmortem FLAIR imaging identified pvWMH in 14/20 specimens and PVI in 5/20 specimens (Figure 1B,C) with sizes ranging from 4.9 to 34 mm^3^. Length of time in fixative was not significantly correlated with any of the histological or immunohistochemical metrics (Table S1). Diagnosis of AD in 10 of the subjects was not significantly associated with any of the histological measures assessed.

**TABLE 1:** Demographic and pathological details of included specimens

Specimen	Age	Sex	Pathology	Fixation (mo.)
1	75	M	Mild AD (Braak stage 1/2)	158
2	71	M	Advanced AD (Braak stage 5/6), NFT, CAA, superficial siderosis	151
3	80	F	Advanced AD (Braak stage 5/6)	155
4	68	F	Mild AD (Braak stage 1/2)	155
5	83	M	Advanced AD (Braak stage 5/6)	160
6	73	F	Advanced AD (Braak stage 5/6), CVD, acute infarct, vasculopathy	161
7	91	F	Advanced AD (Braak 5/6), cerebrovascular disease, atherosclerosis	155
8	84	M	Old infarct cerebellum, mild AD (Braak stage 1/2)	56
9	80	F	Advanced AD (Braak 5/6), CVD	154
10	83	F	Advanced AD (Braak stage 5/6), diffuse Lewy body, CVD, CAA	155
11	78	M	Acute microinfarcts	151
12	75	M	Cerebrovascular disease & old bleed	154
13	78	F	Multiple infarcts	148
14	47	M	Multiple infarcts, CVD, arteriosclerosis	157
15	85	M	CVD, Crookes hyalinization, acute HIE CA1	162
16	65	F	No significant findings	151
17	52	F	No significant findings	158
18	69	M	No significant findings	164
19	70	M	No significant findings	159
20	52	F	No significant findings	161

### 3.1 Microvessel stenosis is associated with periventricular infarction

**FIGURE 2:**
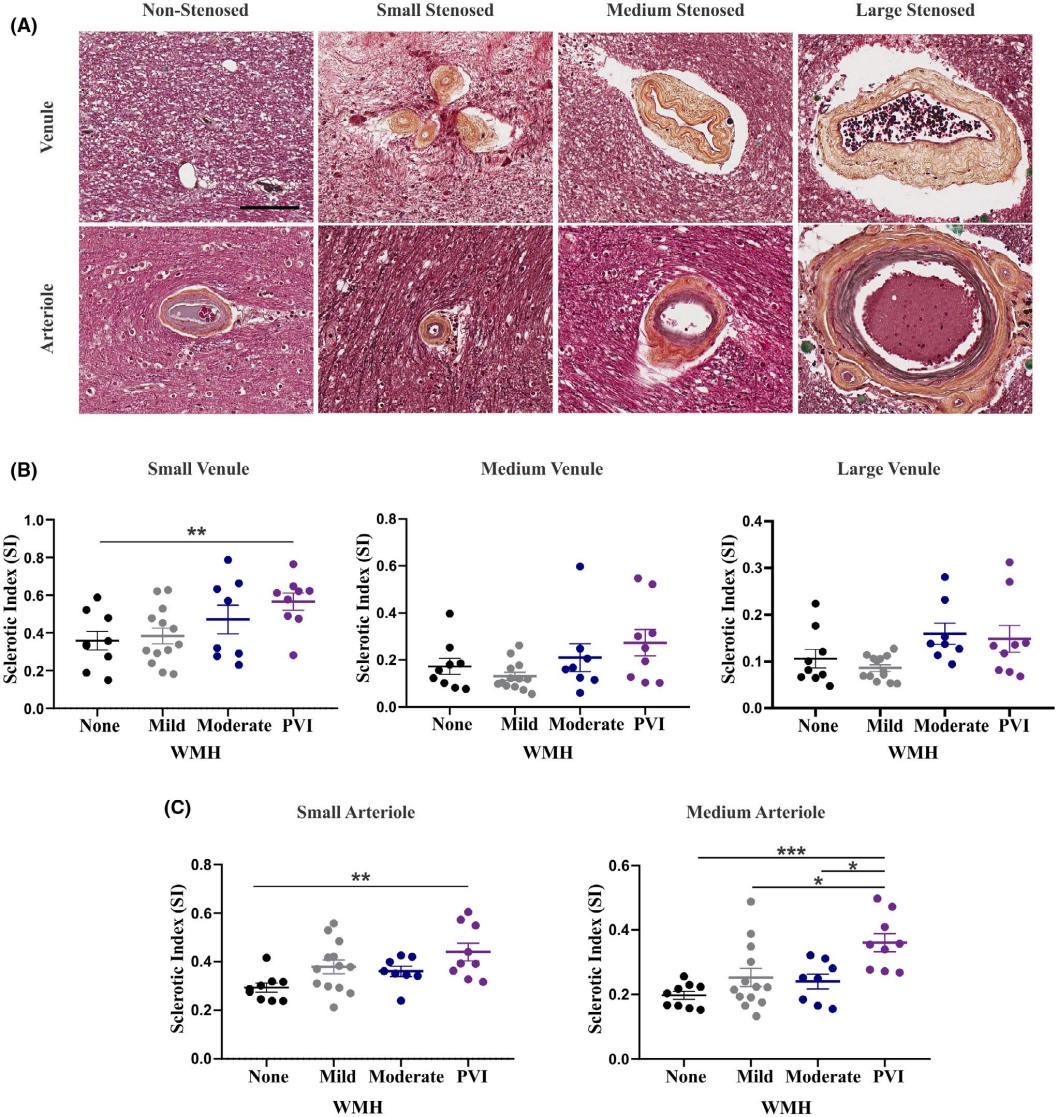
Stenosis of both the small arterioles and venules is evident in the periventricular and subcortical white matter, and the severity is associated with imaging evidence of PVI. (A) Movat's pentachrome staining of arterioles and venules including non‐stenosed (with minimal collagen accumulation), small stenosed (<50 μm diameter), medium stenosed (50–200 μm diameter), and large stenosed (>200 μm diameter). All images captured at 20x magnification; scale bar indicates 100 μm. IHC for SMA was used to distinguish arterioles from venules. (B) Average SI of the small, medium, and large venules quantified in the periventricular white matter of each tissue block in specimens with none, mild, or moderate pvWMH or PVI. (C) Average SI of the small and medium arterioles quantified in the periventricular white matter of each tissue block in specimens with none, mild, or moderate pvWMH or PVI. SI of the small arterioles is increased in specimens with PVI in comparison to those with no WMH (*p *= 0.0063).SI of the medium arterioles is increased in specimens with PVI in comparison to those with no (*p *= 0.0006), mild (*p *= 0.0175), or moderate pvWMH (*p *= 0.0180). Comparison performed using one‐way ANOVA with Tukey's post‐hoc analysis, significance value of *p* = 0.05

The first aim of this study was to describe microvessel stenosis within the white matter of the specimens with pvWMH and PVI. To measure stenosis of the microvessels, the SI of small, medium, and large venules and arterioles within the periventricular white matter and subcortical white matter was assessed (Figure 2A). Qualitatively, drastic reductions in lumen size and visible thickening of collagen within the tunica adventitia of stenosed vessels was evident. When comparing vessels in the two anatomical regions, only the small venules were significantly more stenotic in the periventricular white matter (40.41 ± 3.459) than in the subcortical white matter (22.17 ± 1.824, *p *< 0.0001). We analyzed periventricular stenosis values of small and medium arterioles and small, medium, and large venules from specimens with periventricular infarcts (PVI) in comparison to specimens with no, mild, or moderate pvWMH. When stratifying by size, only the SI of the small periventricular venules remained significantly increased in specimens with PVI (0.566 ± 0.137, *p* = 0.035) overall and in comparison to specimens with no pvWMH (0.359 ± 0.148, *p* = 0.048) (Figure 2B). When grouping the arteries by size, SI of small periventricular arterioles displayed a significant increase in PVI specimens (0.439 ± 0.109, *p* = 0.012) overall and when compared to specimens with no pvWMH (0.293 ± 0.056, *p *= 0.006) (Figure 2C). SI of the medium periventricular arterioles displayed a significant increase in PVI (27.91 ± 2.586, *p *= 0.0262) overall and when compared to specimens without pvWMH (0.197 ± 0.037, *p* = 0.001), with mild pvWMH (0.252 ± 0.102, *p* = 0.018), and moderate pvWMH (0.240 ± 0.065, *p* = 0.018) (Figure 2C). Stenosis of the subcortical venules and arterioles was not significantly associated with the WMH rating or the presence of periventricular infarction.

In addition to SI, we measured the area of the perivascular spaces around the measured arterioles and venules. Within the periventricular white matter, the area of the PVS around small arterioles was significantly increased (2524 µm^2^ ± 1769, *p* = 0.004) overall and when compared to subjects without pvWMH (599.2 µm^2^ ± 469.5, *p* = 0.004), with mild pvWMH (1073 µm^2^ ± 1110, *p* = 0.023), and with moderate pvWMH (1022 µm^2^ ± 430.6, *p* = 0.040).

### 3.2 Fibrinogen uptake occurs within the white matter and is associated with periventricular infarction

**FIGURE 3:**
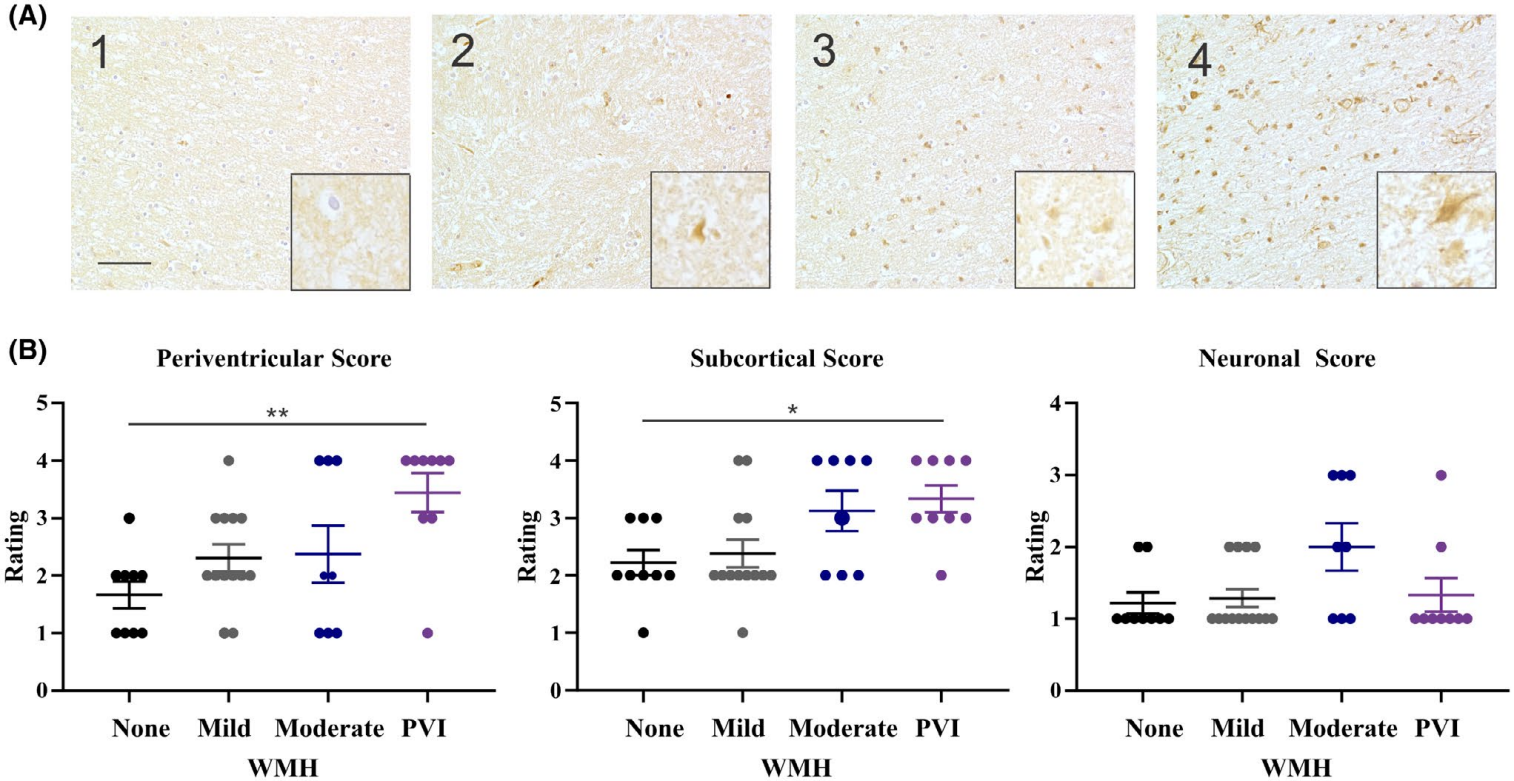
IHC reveals intraglial fibrinogen staining within the periventricular and subcortical white matter is associated with PVI. (A) Fibrinogen rating scale used to quantify accumulations of fibrinogen. Intraglial scale was as follows: 1 = none, 2 = isolated, 3 = frequent but affecting <50% of glial cells, 4 = very frequent affecting >50% of glial cells. Images capturedat 20x with a 40x insert, scale bar indicates 100 μm. (B) Fibrinogen ratings were assigned to images taken within the periventricular or subcortical white matter and cortical neurons (3 images per region, per tissue block were used to generate a rating) in subjects with none, mild, moderate pvWMH or PVI. An increase in periventricular white matter ratings of fibrinogen is associated with PVI (*p* = 0.0076). Significant overall group differences in subcortical white matter ratings of fibrinogen (*p* = 0.0188). No significant differences in neuronal fibrinogen ratings across WMH groups. Comparison performed using Kruskal–Wallis test with Dunn's post‐hoc analysis of multiple comparisons, significance value of *p* = 0.05

To investigate disruptions of BBB function, the presence of fibrinogen within the white matter was characterized. Fibrinogen IHC revealed intraglial, intraneuronal, and interstitial accumulations of fibrinogen which were all assessed independently. Fibrinogen staining was frequently detected within rounded cell bodies of the white matter that had the morphological appearance of oligodendrocytes. Dual labelling with the oligodendrocyte marker Olig2 confirmed the co‐localization of fibrinogen in these cells (Figure S1). There was a smaller population of fibrinogen positive cells with a ramified phenotype more characteristic of microglia or astrocytes, therefore the term intraglial is being used to describe accumulations within the white matter encompassing these three cell populations. Both intraglial fibrinogen in the white matter and intraneuronal fibrinogen within the cortex were assessed using a rating scale (Figure 3A). Age was weakly correlated with the fibrinogen glial rating within the subcortical white matter. (*r* = 0.3318, *p* = 0.039). Interstitial fibrinogen deposition was apparent in specimens with visible accumulations in a halo‐like pattern surrounding vessels of the white matter (Figure S2B). When considering all three types of fibrinogen assessment, only intraglial fibrinogen uptake was associated with PVI. Significantly higher fibrinogen ratings in the white matter were associated with the presence of PVI (*p* = 0.0041) in comparison to individuals without (*p* = 0.0041) and with mild pvWMH (*p* = 0.0187). Regionally, the periventricular white matter fibrinogen ratings were significantly elevated in specimens with PVI in comparison to specimens with no WMH (*p *= 0.0076) (Figure 3B). Overall group differences were significant for subcortical fibrinogen ratings (*p *= 0.0188), without any significant individual differences amongst imaging groups (Figure 3B). Neuronal ratings of fibrinogen uptake did not associate with the presence of WMH or PVI (Figure 3C). Interstitial fibrinogen was not significantly associated with the severity of pvWMH or the presence of PVI.

### 3.3 Periventricular microvessel stenosis is associated with intraglial fibrinogen uptake

Next, we investigated associations between white matter microvessel SI and fibrinogen uptake. Correlations between periventricular arterioles and venules stratified by size category, and corresponding fibrinogen ratings are reported in Table 2. White matter ratings of fibrinogen were significantly correlated with stenosis of the arterioles, both small (*r* = 0.502, *p *= 0.001) and medium (*r* = 0.323, *p *= 0.045), and venules, both medium (*r* = 0.472, *p *= 0.003) and large (*r* = 0.373, *p *= 0.019). Intraneuronal and interstitial fibrinogen pools did not significantly associate with microvessel stenosis of any size.

**TABLE 2:** Microvessel SI is associated with reductions in white matter density and intraglial fibrinogen accumulation

	Periventricular venules			Periventricular arterioles	
	Small	Medium	Large	Small	Medium
Fibrinogen rating	n.s.	*p *= 0.003*	*p *= 0.019*	*p* = 0.001*	*p* = 0.0451*
		*r* = 0.472	*r* = 0.373	*r* = 0.502	*r* = 0.323

Note

Correlations between the SI of venules and arterioles with white matter fibrinogen ratings. Spearman correlations are reported for rating scale analyses, * indicates a correlation with significance value of *p* = 0.05.

### 3.4 Microglial number is associated with fibrinogen uptake in the white matter

**FIGURE 4:**
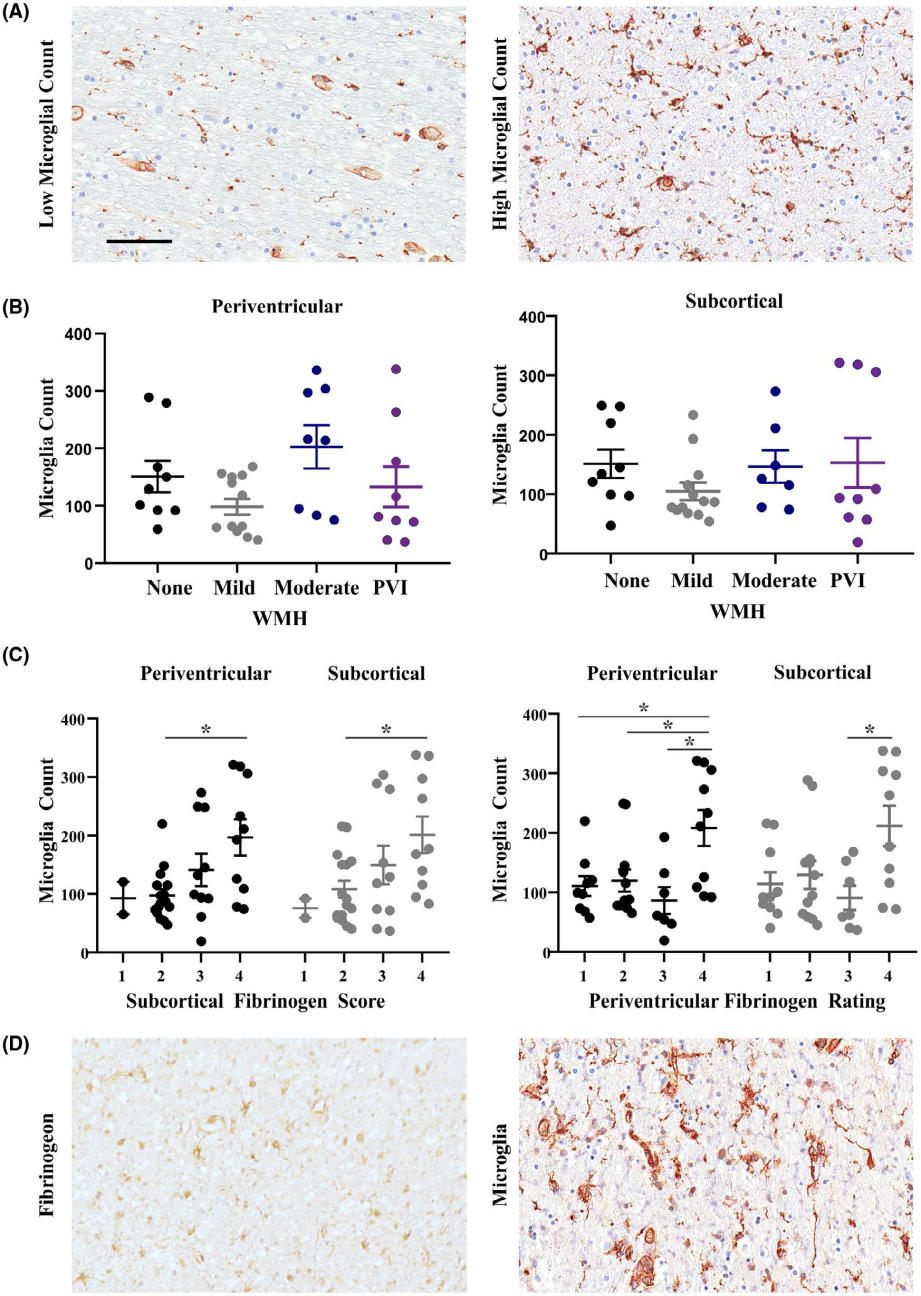
Increases in microglia are seen in the periventricular and subcortical white matter and are associated with fibrinogen uptake. (A) Example images of specimens with high and low levels of microglia, stained with RCA Lectin. (B) Microglia were counted within the periventricular and subcortical white matter (data points represent the average count for 3 images per region) in specimens with none, mild and moderate pvWMH or evidence of PVI. (C) Microglia count within the periventricular and subcortical white matter subjects with fibrinogen ratings of 1–4 in either the periventricular or subcortical white matter. Microglia count is associated with both periventricular and subcortical fibrinogen ratings. Comparison performed using one‐way ANOVA with Tukey's post‐hoc analysis, significance value of *p* = 0.05. (D) Example images taken from the subcortical white matter of a specimen with high fibrinogen rating and high microglial count. All images captured at 20x magnification; scale bar indicates 100 μm

Finally, we sought to characterize increases in microglia cell number in specimens with evidence of fibrinogen uptake. Distinction of microglia using RCA lectin, a histological marker that stains microglia and endothelial cells [36], was effective for the assessment of cell number after further differentiation from endothelia based on size and shape criterion. The number of microglia was quantified within the periventricular and subcortical white matter (Figure 4A). Increases in periventricular microglia number were significantly associated with intraglial fibrinogen ratings within the subcortical (*p *= 0.0153) and periventricular white matter (*p *= 0.0046) (Figure 4C). Similarly, increases in subcortical microglia number were significantly associated with both subcortical (*p *= 0.0329) and periventricular (*p *= 0.0178) fibrinogen ratings (Figure 4C). Microglia number was not significantly associated with pvWMH or PVI (Figure 4B).

## 4 DISCUSSION

The role of WMH in AD, vascular and mixed dementias is becomingly increasingly clear, making it imperative to gain a better understanding of mechanisms that underlie the progression of WMH in these disease [37, 38, 39, 40]. The primary objective of this study was to investigate microvascular disease and blood–brain barrier leakiness in the white matter of specimens with postmortem imaging evidence of SVD. Recent studies have highlighted the utility of postmortem MRI as a reliable method for detecting imaging evidence of SVD that allows for direct spatial comparison between histological and imaging abnormalities [41]. Consistent with previous neuropathological studies, the small vessels in the periventricular white matter displayed reductions in lumen size and thickening of collagen in the vessel wall [34, 42]. As a watershed region, the periventricular white matter does not have a rich arterial blood supply and relies mainly on diffusion of nutrients [43]. This may make this region particularly vulnerable to the effects of hypoperfusion because of arteriole stenosis, where impaired blood delivery contributes to chronic ischemia of the white matter resulting in the rarefaction of white matter tissue [44].

Despite recognition that hypoperfusion and ischemia may cause white matter disease, the mechanistic effects of stenotic vessels on the integrity of the white matter remain unclear. While many factors likely contribute to myelin and axon reduction associated with WMH, the role of microvessel stenosis may be mediated by impairments in BBB function. Extravasation of serum proteins, such as fibrinogen, is a consequence of BBB dysfunction with potentially toxic effects to the cellular environment of the white matter [32]. Recent studies point to age‐related increases in BBB leakiness [29], which can be exacerbated in the context of SVD [23]. Histologically, evidence of fibrinogen leakage was considered in this study by accounting for intraglial, intraneuronal as well as interstitial pools. All three types of deposition were assessed separately since they may not occur at the same rate or have the same consequences. We demonstrate that of the three subtypes of reported fibrinogen uptake, only intraglial accumulation of fibrinogen was associated with histological evidence of stenosis. This is the first study, to our knowledge, demonstrating an association between periventricular stenosis of arterioles with fibrinogen uptake in the white matter in human postmortem subjects. These results are consistent with recent findings suggesting that hypoperfusion because of SVD and BBB dysfunction are related, with endothelial dysfunction and pericyte loss acting as potential mediators [23].

The primary means of assessing SVD clinically is with the use of neuroimaging, therefore in addition to studying the microvessels and fibrinogen uptake histologically, both features were compared to MRI markers of SVD. Both pvWMH and PVI were considered in the analyses as these were evident in postmortem images. Both subcortical and periventricular vessels were analyzed since it has been proposed that vascular changes and BBB disruption related to SVD may extend beyond MRI‐visible lesions [24]. However, only periventricular vessels demonstrated associations with pvWMH or PVI. Stenosis of small and medium periventricular arterioles was associated with the presence of PVI. Evidence of enlarged PVS surrounding small arterioles was also associated with PVI. Enlarged PVS are frequently reported features of SVD and have been recently associated with increased compromise of the BBB [45], however this phenomenon requires further investigation.

Although less frequently studied, venules have also been implicated in the pathogenesis of WMH [22, 34]. In the present study, small venules were the only vessel size to be significantly more stenotic in periventricular white matter in comparison to subcortical white matter and significantly more stenotic in specimens with PVI. Small venules within periventricular white matter might be particularly at risk of becoming occluded following accumulation of collagen within their walls because of their small lumen [34]. The resulting occlusion may prevent adequate drainage through these small venules and result in venous insufficiency and subsequent edema. Although large venules were visibly affected by collagenosis, the SI levels were not associated with PVI in our sample which is in contrast to other analyses of large vein stenosis [22]. Due to their larger lumen, SI values in the large venules of our samples were often lower and this may have reduced the sensitivity of the analysis to the degree of collagenosis.

Despite associations with PVI, stenosis and fibrinogen uptake were not associated with the size of pvWMH (mild vs. moderate). There are other microvessel abnormalities which were not addressed in this study, such as lipohyalinosis and amyloid angiopathy that have been reported in SVD and may also be increased in the presence of pvWMH. Additionally, the lack of severe pvWMH in our sample did not allow us to study microvessel stenosis in specimens with far‐reaching pvWMH that extend deep into the subcortical white matter. It is also possible that the presence of periventricular infarcts was underestimated in this study, as only infarcts present within the same coronal slice as the WMH assessed were accounted for. Therefore, some mild or moderate pvWMH could have had more severe underlying tissue damage if the PVI had not been captured in the coronal section featuring the pvWMH. Furthermore, we did not account for microinfarcts that can be identified pathologically but are often missed on postmortem neuroimaging [46]. Overall, these findings confirm previous reports of microvessel stenosis in association with imaging evidence of pvWMH [47, 48] but suggest that the identification of PVI may be more indicative of severity of stenotic pathology rather than pvWMH alone. pvWMH are often interpreted clinically as lesions of presumed vascular origin, however, the role of axonal pathology, ependymal damage, and AD‐related pathology cannot be excluded with neuroimaging alone especially when there is no evidence of clear ischemic damage (PVI). Given the known heterogeneity in the etiologies of pvWMH, identifying PVI within lesions may be useful clinically in confirming those that are ischemic and of vascular origin.

Consistent with the microvessel stenosis findings, intraglial fibrinogen uptake within periventricular white matter was significantly increased in cases of PVI. Our fibrinogen findings are consistent with previous reports of associations between intraglial and intraneuronal fibrinogen uptake with SVD [26, 27]. The lack of association between fibrinogen and pvWMH may be caused by our mixed diagnostic cohort of specimen, as previous studies have demonstrated that fibrinogen is associated with SVD in normal control specimens but not in Alzheimer's disease [26]. Additionally, preclinical studies have demonstrated age‐related increases in BBB permeability in the hippocampus and medial temporal lobe, but not in the white matter [49, 50, 51]. Therefore, further elucidation of the anatomical regions that are most at risk because of increases in age‐related BBB permeability in the absence of severe SVD or infarction is required. In contrast to the observed intraglial fibrinogen accumulation, we do not report significant associations of intraneuronal or interstitial fibrinogen with SVD imaging markers. Previous studies of serum protein extravasation in SVD have also reported negative findings regarding interstitial fibrinogen deposition [27]. Interstitial staining may represent a transient pool of fibrinogen that is adequately cleared from the white matter, or an earlier state of fibrinogen leakage that precedes damage to the structural integrity of the white matter, consistent with the toxic effects of fibrinogen on the white matter microstructure seen in preclinical models of blood–brain barrier dysfunction [52].

We report that fibrinogen rating is associated with an increase in microglia density. Due to the postmortem nature of this study, the temporality of fibrinogen uptake into the white matter and its potential effect on glial cell activity requires further study. Future *in vitro* modelling can allow for the study of possible microglial increases in response to fibrinogen exposure, either via recruitment or local proliferation.

Microglia number was not significantly associated with microvessel SI, pvWMH or PVI in our specimens. This is in contrast to previous studies which have reported microglia in pvWMH [53] and there are a few potential explanations for this contradiction. First, most studies have previously reported increases specifically in activated microglia, whereas we assessed the total population of microglia using the histological RCA lectin stain instead of accounting for differences in activation state (prevented by prolonged fixation state of the samples). A recent study reported only a weak correlation between total (Iba+) microglia and SI [54]. Furthermore, previous studies have reported that postmortem MRI, in comparison to pathological analysis of myelin pallor, is not as sensitive to white matter changes that contain activated microglia [46]. Our classification of subjects based on white matter changes severe enough to be identified as pvWMH on MRI may have limited our detection of more subtle disease. Finally, while fibrinogen uptake was associated with PVI, microgliosis may not be specific to ischemia and can increase in the white matter under a variety of contexts. Since our selection of brains was heterogeneous in neuropathological diagnosis, it is difficult to rule out other causes of microgliosis seen in the white matter. Future analyses investigating microglia activation may be more specific to ischemic conditions associated with pvWMH and could build upon the associations we report between fibrinogen extravasation and microgliosis.

## 5 CONCLUSIONS

This postmortem study confirms the association between both imaging and histological markers of SVD and extravasation of fibrinogen in a mixed diagnostic sample. The ability to detect SVD during pre‐symptomatic phases in midlife using imaging makes it a promising target for potential intervention. This is the first study, to our knowledge to demonstrate, the association between fibrinogen and microglial number within postmortem human tissue. Further characterization of fibrinogen uptake in the white matter, and its association with imaging markers of SVD, is necessary to understand potential effects on glial cells and the maintenance of white matter integrity. Due to the co‐morbidity of SVD with aging and neurodegenerative diseases, understanding cellular changes associated with disease progression can identify important therapeutic targets for the prevention of cognitive decline.

## CONFLICT OF INTEREST

The authors declare no conflict of interest.

## ETHICS APPROVAL

Informed consent was obtained for all specimens, according to the Declaration of Helsinki.

## AUTHOR CONTRIBUTIONS

Austyn D. Roseborough: study design, neuroimaging analysis, tissue dissection, microscopic assessments, image analysis, statistical analysis, manuscript drafting, and preparation. Berk Rasheed, Youngkyung Jung, William Pinsky: study design, neuropathology, critical review of the manuscript. Kristopher D. Langdon, Robert Hammond: study design, neuropathology, and critical review of the manuscript. Stephen H. Pasternak: study design, critical review of the manuscript. Ali R. Khan: neuroimaging analysis, study design, and critical review of the manuscript. Shawn N. Whitehead: study design, microscopic assessments, and critical review of the manuscript.

## Supporting information

Fig S1
**FIGURE S1** Dual labelling of fibrinogen and Olig2 or NeuN. (A) IHC confirms co‐localization of fibrinogen (brown) and Olig2 (pink) within the white matter. (B) IHC confirms co‐localizations of fibrinogen (brown) and NeuN (pink) in the grey matter. Images taken at 20x magnification; scale bar indicated 100 μm

Fig S2
**FIGURE S2** Interstitial fibrinogen staining is seen in the parenchyma surrounding white matter vessels. (A) Vessel with minimal fibrinogen extravasation, (B) vessel with apparent halo of fibrinogen in surrounding tissue. Images taken at 20x scale bar indicates 50 μm

Table S1
**TABLE S1** Correlations between time in fixative and histological or immunohistochemical metrics used in analyses

## Data Availability

The data that support the findings of this study are available from the corresponding author upon reasonable request.
