# Supplementary figures and images for: Microvessel stenosis, enlarged perivascular spaces, and fibrinogen deposition are associated with ischemic periventricular white matter hyperintensities
Source: Brain Pathol. 2021 Sep 19;32(1):e13017. doi: 10.1111/bpa.13017 (PMC8713528; doi:10.1111/bpa.13017)

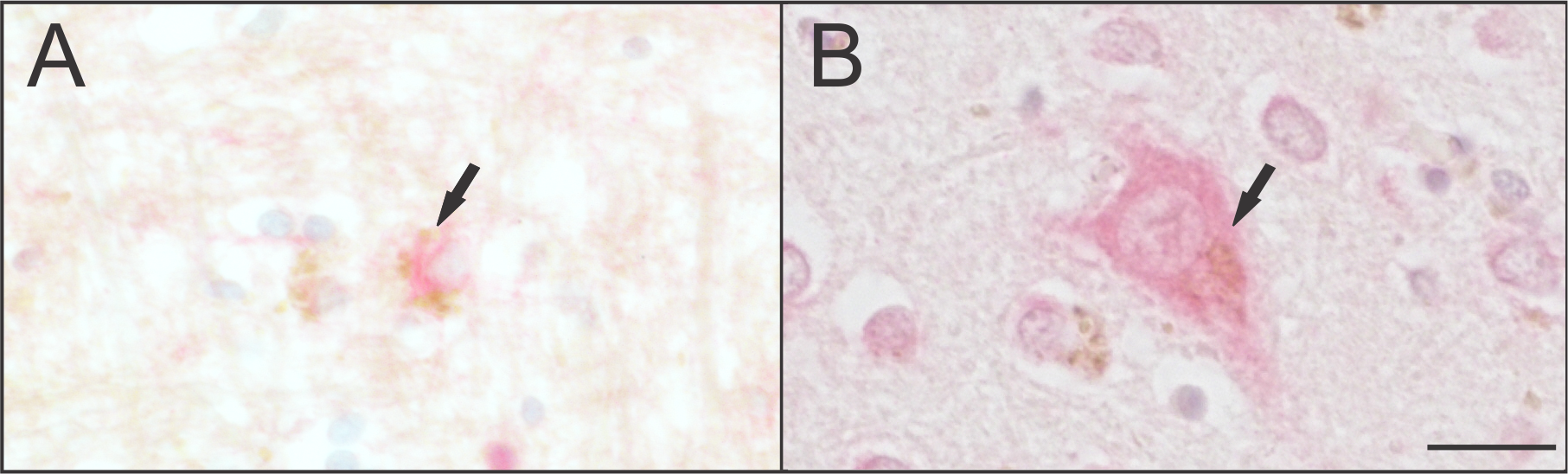

Supplement: Supplementary file 1 — Fig S1 FIGURE S1 Dual labelling of fibrinogen and Olig2 or NeuN. (A) IHC confirms co‐localization of fibrinogen (brown) and Olig2 (pink) within the white matter. (B) IHC confirms co‐localizations of fibrinogen (brown) and NeuN (pink) in the grey matter. Images taken at 20x magnification; scale bar indicated 100 μm [file BPA-32-e13017-s001.tif]
