# Supplementary material for: Microvessel stenosis, enlarged perivascular spaces, and fibrinogen deposition are associated with ischemic periventricular white matter hyperintensities
Source: Brain Pathol. 2021 Sep 19;32(1):e13017. doi: 10.1111/bpa.13017 (PMC8713528; doi:10.1111/bpa.13017)
Supplement: Supplementary file 3 — Table S1 TABLE S1 Correlations between time in fixative and histological or immunohistochemical metrics used in analyses [file BPA-32-e13017-s002.docx]

**TABLE S1 Correlations between time in fixative and histological or immunohistochemical metrics used in analyses**

| **Measurement** | **r** | **p-value** |
| --- | --- | --- |
| Fibrinogen % area | -.274 | .123 |
| Fibrinogen Periventricular Rating | -.308 | .082 |
| Fibrinogen Subcortical Rating | -.197 | .272 |
| Fibrinogen Cortical Rating | .184 | .306 |
| Microglia Count | .120 | .507 |
| LFB % Area | -.093 | .607 |
| NF % Area | -.116 | .520 |
